# The Arg282Ser missense mutation in APOA5 gene determines a reduction of triglyceride and LDL-cholesterol in children, together with low serum levels of apolipoprotein A-V

**DOI:** 10.1186/s12944-017-0569-4

**Published:** 2017-09-19

**Authors:** Laura Bertoccini, Federica Sentinelli, Michela Incani, Diego Bailetti, Flavia Agata Cimini, Ilaria Barchetta, Maria Gisella Cavallo, Efisio Cossu, Andrea Lenzi, Sandro Loche, Marco Giorgio Baroni

**Affiliations:** 1 Department of Experimental Medicine, Sapienza University of Rome, Policlinico Umberto I, 00161 Rome, Italy; 2 Endocrinology and Diabetes, Department of Medical Sciences, University of Cagliari, Cagliari, Italy; 3 Pediatric Endocrine Unit, Regional Hospital for Microcitemia, Cagliari, Italy; 4 Neuromed, Pozzilli, IS Italy

**Keywords:** APOA5 variant, Lipids, Children, Obesity, Apolipoproteins, Triglycerides

## Abstract

**Background:**

Apolipoprotein A-V (ApoA-V) is a recognized regulator of plasma triglycerides (TGs), and previous studies have shown associations between variants in *APOA5* (apolipoprotein-A5) gene and high TG levels. Recently, a new association between the Arg282Ser missense mutation (rs778114184 G > T) in *APOA5* gene and decreased triglyceride levels has been shown in an adult population from Sardinia.

In this study we add further insight into the role of *APOA5* by exploring whether this association begins early in life in children, or becomes manifest only in adulthood. We performed the genetic association analysis of *APOA5* in a cohort of 925 overweight and obese children and adolescents from Sardinia, Italy, to see if the genetic burden is already at play before modifying risk factors are interacting.

**Results:**

We identified 24 heterozygous subjects for the Arg282Ser variant and no homozygous subject. Here we show that the Arg282Ser mutation in *APOA5* gene is associated with a significant reduction of TG (−15.5 mg/dl), total (−18.1 mg/dl) and LDL-cholesterol (−14.8 mg/dl) levels in overweight/obese children and adolescents, indicating that indeed this association appears early in life. Also, we observed a significant reduction in serum apoA-V levels in heterozygous children.

**Conclusions:**

Our data clearly show that the Arg282Ser mutation in *APOA5* gene determines a reduction of TG, total and LDL-cholesterol and apolipoprotein A-V levels in overweight/obese children and adolescents, demonstrating that this mutation has the power to affect lipid levels already since childhood.

## Background

Lipid concentrations are heritable traits. Population and family studies suggest that lipid levels have a genetic component with heritability estimates for triglycerides (TGs), total cholesterol, LDL and HDL-cholesterol varying between 48 to 59% [1].

Recently Sidore et al. [2] described, by whole-genome sequencing in 6602 subjects from Sardinia, Italy, a new association between the missense mutation Arg282Ser (rs778114184) in *APOA5* gene and decreased triglyceride (TG) levels.

Moreover, the authors reported that this is the strongest variant modulating triglycerides levels in Sardinia, explaining almost 1% of the phenotypic variance.

Aside from the Sardinian population, the Arg282Ser variant has been found in only two chromosomes in >30.000 Europeans characterized in the Exome Aggregation Consortium [2].

The *APOA5* gene (apolipoprotein-A5) is located on human chromosome 11 at position 11q23. The gene for apolipoprotein A-V (apoA-V) is small, spanning approximately 3 kbp, is composed of 4 exons and 3 introns and codes for the 366 amino acid protein apoA-V. In humans, *APOA5* is expressed almost exclusively in the liver tissue [3]; some minor expressions have also been detected in the small intestine [4].

ApoA-V has been identified as an important determinant of plasma TG levels in humans and mice since its discovery [3, 5]. An inverse correlation between plasma apoA-V and TG levels has been reported from animal studies [3, 6], which has led to the idea that plasma apoA-V levels would correlate negatively with plasma TG levels in humans. However, recent studies in human subjects using semi-quantitative immunoassays do not support such a relationship [7], showing instead a positive correlation between apoA-V levels and TG levels. ApoA-V may influence plasma triglyceride levels by different possible mechanisms, including effects on lipoprotein lipase [8], on VLDL secretion [9] and on hepatic uptake of lipoprotein remnants [10]. Despite these possible effects, the mechanism of apoA-V action, particularly in humans, remains unclear.

Several SNPs within the *APOA5* gene have been associated with changes in plasma TG levels. Population frequencies of common *APOA5* alleles exhibit large interethnic differences. For example, there are about 15% of carriers of the −1131C allele of rs662799 variant among Caucasians, but the frequency could reach even 40% to 50% among Asians, although the strongest effect on plasma TG levels has been observed in Hispanics [11].

More than twenty rare variants have been described within the human *APOA5* gene [11–14]. They cover a wide spectrum that includes stop codons, amino acid changes, as well as insertions and deletions. These mutations are generally associated with hypertriglyceridemia, but penetration is usually not 100% [15]. The missense mutation Arg282Ser (rs778114184) in *APOA5* gene is seemingly the only one associated with decreased triglyceride levels [2].

Since the association between the Arg282Ser mutation in *APOA5* gene and TG levels has been evaluated and observed only in an adult population (mean age of 43.5) [2], we performed a genetic association analysis in a cohort of overweight and obese children and adolescents from Sardinia, Italy. Our aim was to provide further insight into the role of *APOA5* gene by exploring whether the association between the Arg282Ser variant and TG levels is present in this young cohort, when modifying factors may have had only a short time to act, in order to establish whether the genetic predisposition towards reduced TG levels begins early in life.

## Methods

### Study subjects

For this study, a total of 925 overweight/obese children (438 males and 487 females) were recruited from the outpatient clinic of the Pediatric Endocrine Unit for excess body weight, at the Regional Hospital for Microcitaemia in Cagliari, Italy. Presence of endocrine disorders or genetic syndromes was considered as exclusion criteria. None of the subjects were taking any form of medication. Anthropometric, demographic and clinical data were collected at time of enrolment as described before [16]. Body mass index standard deviation score (SDS-BMI) was defined according to Italian growth charts in people aged 2–20 years. SDS-BMI >1 and >2 were used to define overweight and obesity, respectively [17]. Pubertal development stages were determined in conformity with Tanner scale and subjects were divided into two groups: pre-pubertal (Tanner’s stage I) and pubertal (Tanner’s stages II–V).

The University Ethical Committee approved the study (ref n. 45/08/CE), and informed written consent was obtained from the children or their legal guardians.

### Laboratory determinations

Blood glucose, insulin, total cholesterol, LDL- and HDL-cholesterol, triglycerides, alanine aminotransferase (ALT) and aspartate aminotransferase (AST) were measured in all overweight and obese children in the fasting state. Systolic and diastolic blood pressure was measured three times after a 10 min rest and the mean value was used in subsequent analyses.

Plasma glucose was determined by the glucose oxidase method (Autoanalyzer, Beckman Coulter, Fullerton, CA). Plasma insulin concentration was measured on frozen samples using a radioimmunoassay (DLS-1600 Insulin Radioimmunoassay Kit, Diagnostic System Laboratories Inc., Webster, TX) with an intra-assay coefficient of variation (CV) between 4.7% and 12.2% and an inter-assay CV between 4.5% and 8.3%.

ApoA-V levels were measured by a non-selective enzyme-linked immunosorbent assay (ELISA) kit (Aviva System Biology, San Diego CA) on sera frozen immediately after separation and stored at −80 °C. The intra and mean inter-assay precision was ≤3.6% and ≤7.9% respectively as reported by the manufacturer.

### Surrogate indices of insulin-resistance and secretion

HOMA-IR (homeostatic model assessment of insulin resistance) for insulin-resistance assessment, HOMA-B (homeostatic model assessment for beta-cell function) for insulin secretion evaluation were calculated as previously shown by Matthews et al. [18].

### Genotyping assay

The rs778114184 G > T variant was detected by PCR amplification and high resolution melting analysis (HRM) on an Eco™ Real-Time PCR System by Illumina. Fragments were amplified in a reaction volume of 10 μL with 0.5 μM of each primer, 10 ng of genomic DNA, and 1× LCGreen Plus + Melting Dye. After an initial polymerase activation step at 95 °C for 3 min, amplification was performed using 42 cycles of denaturation (95 °C for 15 s) and annealing (60 °C for 1 min). After amplification, a final melting curve was recorded by heating to 95 °C for 15 s, cooling to 65 °C for 15 s, and holding until 95 °C. Mutation carriers were sequenced for validation.

To ensure long-term reproducibility of the method in each experiment, two control samples carrying GG and GT genotypes were run each time.

### Statistical analysis

All statistical analyses were performed with SPSS 17.0 statistical package. Skewed variables were logarithmically transformed before the analyses. Categorical variable distribution was compared by χ2 test. Differences between continuous variables across the genotype classes were evaluated by ANOVA. *P* values were calculated using a linear regression model including gender, age, BMI-SDS, and Tanner stage.

Power calculation: given a standard deviation in TG levels in our population of 35 mg/dl [19] the size of the study cohort had the power of 80% to detect, with an alpha error of 0.05, a mean difference of 20.7 mg/dl [2] in triglyceride levels across different genotypes.

## Results

Among the 925 children studied we identified 24 heterozygous subjects for the rs778114184 variant and no homozygous subject. The genotype frequencies were in Hardy-Weinberg-Equilibrium.

We then analysed the association between the polymorphism and clinical and biochemical characteristics (Table 1).

**Table 1:** Clinical characteristics of overweight and obese children and adolescents stratified by ApoA5 genotype

	Genotypes		*P*
	GG n. = 901	GT n. = 24	
Females/Males*	477/424	10/14	0.380
Age (years)	10.4 ± 3.2	9.9 ± 3.2	0.500
Weight (Kg)	57 ± 19.8	55 ± 18.5	0.602
BMI (Kg/m^2^)	27.4 ± 4.2	29.1 ± 9.3	0.216
SDS-WEIGHT	1.5 ± 0.9	1.5 ± 0.8	0.855
SDS-BMI	2.8 ± 1.2	3.6 ± 2.9	0.103
SBP (mm/Hg)	106 ± 14.8	105 ± 14.6	0.811
DBP (mm/Hg)	61.7 ± 8.8	63 ± 8.7	0.515
TC (mg/dl)	167.8 ± 32.3	149.7 ± 25.3	0.005
TG (mg/dl)	63.7 ± 39.5	48.2 ± 31.6	0.001
HDL-C (mg/dl)	51.5 ± 12.2	51.2 ± 11.1	0.996
LDL-C (mg/dl)	103.4 ± 28.2	88.6 ± 22.6	0.016
AST (U/L)	24.4 ± 10.7	25.6 ± 5.9	0.591
ALT (U/L)	23 ± 12.5	22.6 ± 7.8	0.873
BG 0′ (mg/dl)	89 ± 7.6	88.2 ± 6.3	0.656
BG 120′ (mg/dl)	105.5 ± 20.2	113.2 ± 17	0.055
Ins 0′ (μUI/ml)	14.7 ± 9.1	17.9 ± 12.4	0.376
Ins 120′ (*μ*UI/ml)	61.4 ± 51	90.3 ± 73.5	0.087
HOMA-IR (U)	3.3 ± 2.1	4 ± 2.8	0.421
HOMA-B	218.3 ± 183	257.3 ± 177.6	0.314

Values are expressed as means ± standard deviations

Abbreviations: *BMI* body mass index, *SDS* Standard Deviation Score, *SBP* systolic blood pressure, *DBP* diastolic blood pressure, *TC* total cholesterol, *TG* plasma triglycerides, *HDL-C* high density lipoprotein-cholesterol, *LDL-C* low density lipoprotein cholesterol, *AST* aspartate aminotransferase, *ALT*, alanine aminotransferase, *BG* blood glucose, *Ins* insulin, *HOMA-IR* homeostatic model assessment of insulin resistance, *HOMA-B* homeostatic model assessment for beta-cell function U, Unit

*P value was calculated by χ2. *P* values <0.05 are considered significant

**Fig. 1:**
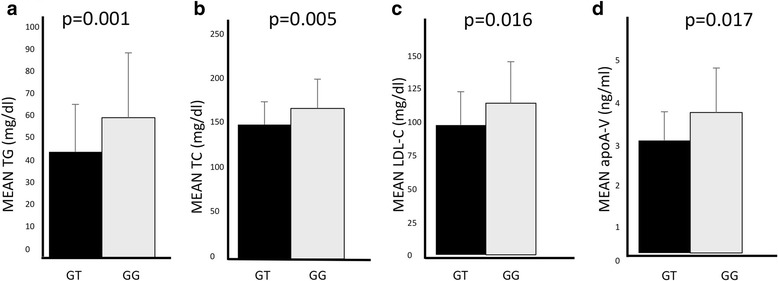
Association between *ApoA5* genotypes and lipid profile in a cohort of overweight and obese children and adolescents. Mean plasma levels of (**a**) triglycerides (TG), (**b**) total cholesterol (TC) and (**c**) LDL- cholesterol (LDL-C) stratified by ApoA5 genotypes. *P* values were calculated using linear regression including age, gender, BMI and Tanner stage as covariates. Mean serum levels of (**d**) ApoA5 protein in a subgroup of 24 heterozygous and 20 wild-type children stratified by ApoA5 genotypes

Carriers of the mutation showed significantly reduced levels of TGs (GT vs GG, 48.2 ± 31.6 vs 63.7 ± 39.5, *p* = 0.001), total cholesterol (GT vs GG, 149.7 ± 25.3 vs 167.8 ± 32.3, *p* = 0.005), and LDL-cholesterol (GT vs GG, 88.6 ± 22.6 vs 103.4 ± 28.2, *p* = 0.016) (Fig. 1 a, b and c). Thus, the mutation Arg282Ser (rs778114184 G > T) in *APOA5* gene is associated with a reduction of TG (−15.5 mg/dl), total (−18.1 mg/dl) and LDL-cholesterol (−14.8 mg/dl) levels in overweight/obese children and adolescents.

Additionally, multiple linear regression analysis was performed to estimate which variables were independently associated with serum triglycerides (Table 2). We observed that BMI (*p* < 0.001), Arg282Ser mutation (p < 0.001), and HOMA-IR (p < 0.001) were significantly and independently associated with serum triglyceride levels. Also, with the same model, a significant independent association was observed between the *APOA5* Arg282Ser mutation and total- (Table 3) and LDL- cholesterol (Table 4).

**Table 2:** Multiple linear regression analysis

Independent Variable	Standardized Regression Coefficients	t value	*P*
Age	0.029	0.628	0.530
BMI	0.153	3.761	<0.001
Gender	0.015	0.458	0.647
TANNER’S STAGE	−0.017	−0.379	0.705
Arg282Ser SNP	−0.134	−4.229	<0.001
HOMA-IR	0.192	5.513	<0.001

Triglycerides are the dependent variable

Abbreviations: *BMI* body mass index, *HOMA-IR* homeostatic model assessment of insulin resistance

*P* values <0.05 are considered significant

**Table 3:** Multiple linear regression analysis

Independent Variable	Standardized Regression Coefficients	t value	*P*
Age	0.015	0.311	0.756
BMI	−0.022	−0.522	0.602
Gender	0.045	1.337	0.182
TANNER’S STAGE	−0.125	−2.754	0.006
Arg282Ser SNP	−0.091	−2.743	0.006
HOMA-IR	0.058	1.597	0.111

Total cholesterol is the dependent variable

Abbreviations: *BMI* body mass index, *HOMA-IR* homeostatic model assessment of insulin resistance

*P* values <0.05 are considered significant

**Table 4:** Multiple linear regression analysis

Independent Variable	Standardized Regression Coefficients	t value	*P*
Age	0.007	0.133	0.894
BMI	−0.44	−1.039	0.299
Gender	0.025	0.738	0.460
TANNER’S STAGE	−0.090	−1.966	0.050
Arg282Ser SNP	−0.076	−2.294	0.022
HOMA-IR	0.102	2.809	0.005

LDL- cholesterol is the dependent variable

Abbreviations: *BMI* body mass index, *HOMA-IR* homeostatic model assessment of insulin resistance

P values <0.05 are considered significant

Next, we measured serum apolipoprotein A-V concentration in the 24 carriers of the rs778114184 variant and in a group of wild-type children, matched for sex, age and BMI; our aim was to test if there was a difference in apoA-V levels between carriers and non-carriers of the Arg282Ser mutation in *APOA5* gene.

We observed that serum ApoA-V levels were significantly lower in heterozygous than wild type subjects (3.07 ± 0.78 vs 3.83 ± 1.22 ng/ml, *p* = 0.017) (Fig. 1 d) demonstrating a direct relation between TG and apoA-V levels.

## Discussion

Apolipoprotein A-V is a key regulator of plasma triglycerides. Here we show that the mutation Arg282Ser (rs778114184 G > T) in *APOA5* gene is associated with a reduction of TG, total and LDL-cholesterol levels in overweight/obese children and adolescents, showing that this association appears early in life and that the genetic burden is already at play before modifying risk factors interact. In agreement with the observation from Sidore et al. [2] we also observed that the Arg282Ser mutation in *APOA5* gene explained almost 1% of the total variance in TGs in our cohort, further underlining the relevant role played by this variant in the genetic determination of the trait.

These results were confirmed by multivariate regression analysis, where a negative independent association was observed for the Arg282Ser variant in *APOA5* gene. In addition, we observed a significant reduction in serum apoA-V levels in heterozygous children, highlighting a direct relation between low apoA-V concentration and low TGs.

Interestingly, the concept that apoA-V and TGs are directly related is supported by several epidemiological studies [7, 20–23]. Indeed, the notion that apoA-V and triglycerides are positively related is supported by findings by Dallinga-Thie et al. [20] in patients with type 2 diabetes, by Vaessen et al. [21] in more than 3000 individuals and by Henneman et al. [22] in patients with severe hypertriglyceridemia. In addition, the direct relationship between apoA-V and TG levels observed in the current analysis further agrees with previous studies by Schaap et al. [7] in patients with markedly elevated TGs and by Talmud et al. [23] in patients with type 2 diabetes.

These observations are in contrast with experiments in animal models, where an inverse correlation between apoA-V concentrations and plasma TG concentrations was detected [7]. The reason for the positive correlation between apoA-V and TGs in humans and the negative correlation in mouse models is unclear, but it suggests that, for apoA-V, inferring from animal models to man should be made with caution [23].

The study performed by Schaap et al. [7] for example provides evidence for a complex interaction between apoA-V and apoC-III. The relative amounts of apoA-V and apoC-III in plasma, and most likely their distribution over lipoproteins, may influence LPL activity and ultimately TG levels [7]. It is evident the need for additional studies to clarify the role of apoA-V in human TGs metabolism.

With regards to the association between *APOA5* and total and LDL-cholesterol, our observation is in line with a previous report showing the same association in GWAs [24]. Which mechanism is involved is a matter of speculation, since to the best of our knowledge, there are no studies exploring this relationship. We could speculate that, by reducing VLDL levels, all the following steps (IDL, remnants) leading to LDL formation from VLDL might be affected.

Strengths of our study rely on the collection of a very well clinically characterized cohort of children from Sardinia, where the Arg282Ser mutation is more common [2] compared to other populations. Moreover, genetic studies performed in children allow reducing the effect of confounding factors, such as diet, physical activity and lifestyle, on the trait under study.

The main limitation of this study is its cross-sectional design, which does not allow establishing a causal link; longitudinal studies are warranted to clarify this question.

## Conclusion

In conclusion, our data clearly show that the Arg282Ser mutation (rs778114184 G > T) in *APOA5* gene is associated with a reduction of TG, total and LDL-cholesterol levels in overweight/obese children and adolescents, demonstrating that this mutation has the power to affect lipid levels already since childhood, when other key modifying factors (i.e. lifestyle, diet, physical activity) may have not yet influenced the traits. In addition, we observed a significant reduction in serum apoA-V levels in heterozygous children with lower TG levels, highlighting the direct relation between apoA-V concentration and TGs, as previously shown in large population studies. Given these effects, apoA-V may possibly represent a putative target for drug development.

## Abbreviations

ALT: Alanine aminotransferase
APOA5: Apolipoprotein-A5
APOA-V: Apolipoprotein A-V
AST: Aspartate aminotransferase
BG: Blood glucose;
BMI: Body mass index
DBP: Diastolic blood pressure
HDL-C: High density lipoprotein-cholesterol
HOMA-B: Homeostatic model assessment for beta-cell function
HOMA-IR: Homeostatic model assessment of insulin resistance
HRM: High resolution melting analysis
INS: Insulin
LDL-C: Low density lipoprotein cholesterol
SBP: Systolic blood pressure
SDS: Standard Deviation Score
SDS-BMI: Body mass index standard deviation score
TC: Total cholesterol
TGs: Triglycerides
